# Utilizing Fast Spin Echo MRI to Reduce Image Artifacts and Improve Implant/Tissue Interface Detection in Refractory Parkinson's Patients with Deep Brain Stimulators

**DOI:** 10.1155/2014/508576

**Published:** 2014-02-25

**Authors:** Subhendra N. Sarkar, Pooja R. Sarkar, Efstathios Papavassiliou, Rafael R. Rojas

**Affiliations:** ^1^Department of Radiology, Beth Israel Deaconess Medical Center, Harvard Medical School, 330 Brookline Avenue, Boston, MA 02215, USA; ^2^School of Medicine, University of Texas Health Sciences Center, San Antonio, TX 78229, USA; ^3^Division of Neurosurgery, Beth Israel Deaconess Medical Center, Harvard Medical School, 330 Brookline Avenue, Boston, MA 02215, USA

## Abstract

*Introduction*. In medically refractory Parkinson's disease (PD) deep-brain stimulation (DBS) is an effective therapeutic tool. Postimplantation MRI is important in assessing tissue damage and DBS lead placement accuracy. We wanted to identify which MRI sequence can detect DBS leads with smallest artifactual signal void, allowing better tissue/electrode edge conspicuity. *Methods*. Using an IRB approved protocol 8 advanced PD patients were imaged within MR conditional safety guidelines at low RF power (SAR ≤ 0.1 W/kg) in coronal plane at 1.5T by various sequences. The image slices were subjectively evaluated for diagnostic quality and the lead contact diameters were compared to identify a sequence least affected by metallic leads. *Results and Discussion*. Spin echo and fast spin echo based low SAR sequences provided acceptable image quality with comparable image blooming (enlargement) of stimulator leads. The mean lead diameters were 2.2 ± 0.1 mm for 2D, 2.1 ± 0.1 mm for 3D, and 4.0 ± 0.2 mm for 3D MPRAGE sequence. *Conclusion*. Low RF power spin echo and fast spin echo based 2D and 3D FSE sequences provide acceptable image quality adjacent to DBS leads. The smallest artifactual blooming of stimulator leads is present on 3D FSE while the largest signal void appears in the 3D MPRAGE sequence.

## 1. Introduction 

In medically refractory Parkinson's disease, deep brain stimulation (DBS) is often an effective therapeutic tool as noted by Obeso et al. [1] acting on the cells and fibers located closest to the implanted electrode. However, it remains unclear exactly how DBS therapy improves symptoms in Parkinson's patients, and the benefits of DBS are currently understood only empirically Okun [2]. Around the lead track, a thin capsule of connective tissue (thickness 5 to 25 *μ*m) is formed surrounded by a 500 *μ*m or less rim of fibrillary gliosis as noted by Haberler et al. [3] after postmortem examination. In the adjacent brain tissue of thickness less than 1 mm, loosely scattered glial fibrillary acidic protein-positive protein astrocytes are found and stimulation seems to modify the tissue microstructure of the local encapsulation, increasing conductivity and decreasing electrode impedance. It is important to directly visualize or indirectly estimate the size and relative contrast of surrounding tissue, preferably by MRI.

The goals of this study were twofold: first we wanted to see if MRI detectable leads have large size difference for patients immediately after implantation with no observable complications, and, second, we sought to explore if there are MR sequence specific size differences in visualized signal voids for DBS leads. The role of MRI for assessing DBS lead placement accuracy has already demonstrated [4] significant advantages despite ignoring the lead-tissue interface.

DBS leads are built with conducting metals (Pt/Ir alloy) that are mildly paramagnetic and produce artifactual signal loss *in vivo* for all MRI sequences. We asked the questions: what the limit of edge detection and localizability of DBS leads is, and which sequence minimizes the blooming artifacts. In other words, it is important to improve visualization of tissue edges adjacent to implant tips and minimize tissue signal loss due to high susceptibility of metallic components in the implanted electrodes.

During an MRI the DBS electrodes deposit a significant amount of radiofrequency induced heat due to the metallic composition (represented by specific absorption rate, SAR) to the surrounding tissue as described by Zrinzo et al. [5] and Tagliati et al. [6]. Hence the DBS vendor Medtronic Inc. and the FDA have provided MR conditional guidance [7, 8] for imaging hardware and a maximum SAR level allowed for imaging of patients with DBS. The present work has followed these restrictions during the development and testing phases on non-DBS subjects and the results have been reported elsewhere by Sarkar et al. [9].

## 2. Materials and Methods 

### 2.1. Patient Selection

Following institutional ethics and research review committee guidelines at our institution, eight patients with advanced Parkinson's disease that were refractory to medications were studied after implantation of DBS for lead localization (4 males, 4 females, age range 53–75 years, disease duration 8–16 years, median 11.5  years, and UPDRS score: more than 30 without medication; medical history included dyskinesia, speech and/or memory difficulty, depression, and gait disorder).

### 2.2. Imaging Details

MR sequences used were 2D fast spin echo T2 (FSE T2) for 4 patients, 3D FSE T2 for 2 patients, Spin echo T1 (SE T1) for 2 patients, and magnetization prepared rapid acquisition gradient echo T1 (MPRAGE T1) for all 8 patients. However, for DBS patients the sequences were prepared so as to run at a low RF power (at SAR < 0.1 W/kg). Following earlier reports [9], we have stretched the refocusing RF pulses by 2–4-fold and reduced the refocusing angles substantially to attain a whole head RF power level of 0.1 W/kg as required per vendor and FDA guidelines [7, 8]. Two readers (EP and RR, both with more than 10 years of clinical experience) judged the imaging efficacy and clinical quality of low SAR images.

In addition to the T2 sequences, two other sequences with spin echo origin (FLAIR T2 and SE T1) were used at low SAR. The FLAIR sequence was qualitatively assessed for infection or other indications and was not used for DBS lead size measurements. Traditionally an inversion-prepared GRE-based high resolution sequence (MPRAGE) is used to image gray/white matter and the DBS leads at a high resolution (typically 1 × 1 × 1 mm^3^ isotropic or 1 × 1 × 1.5 mm^3^  voxel resolution). Being a gradient echo based method, this sequence is sensitive to susceptibility and causes a greater amount of signal loss surrounding the metallic leads. We used the lead diameter from MPRAGE as the maximum artifact size that may be acceptable for visualizing adjacent gray or white matter.

### 2.3. Image-Based Measurements and Statistical Analysis

**Figure 1:**
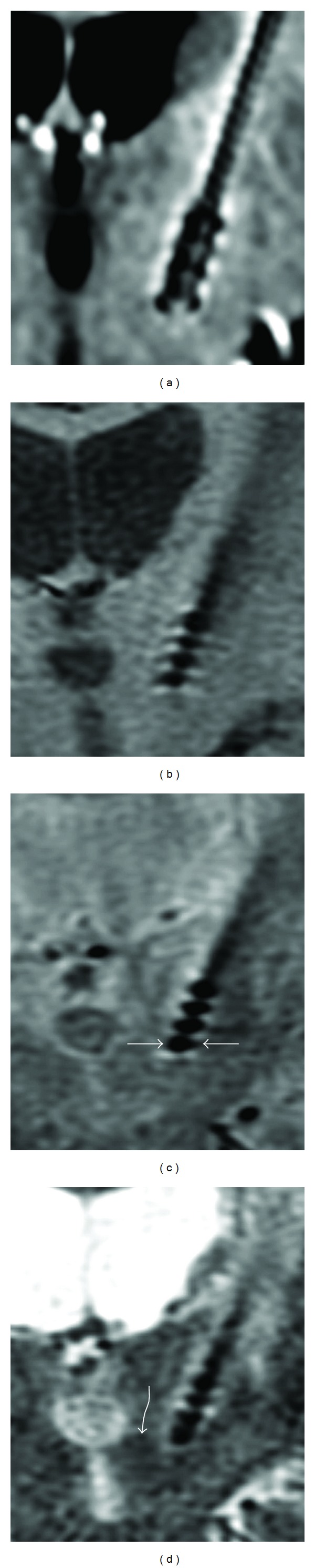
Typical coronal MR images, (a) a section from 3D MPRAGE; measured contact diameter (measurement plan is shown in panel (c) by straight arrows) = 4 mm, more than that from (b) a section from 2D SE T1 (2.2 mm) or (c) 2D FSE PD (2.3 mm) and (d) 2D FSE T2 (2.3 mm) sequences. The location of the lead at left subthalamic nucleus is indirectly estimated as 11-12 mm lateral to midline across the superior-anterior border of the red nucleus (curved arrow, panel (d)).

Each of the DBS leads has 4 contacts (1.5 ± 0.1 mm in height and 1.3 ± 0.1 mm in diameter, separated by plastic sheaths). The diameters of one or more contacts in their largest dimensions were measured from magnified MR images as shown in Figure 1(c). A consistent radiologic intensity window/level was used for all measurements by a single reader, SS. From all 8 patients, one estimate of lead diameter per patient was measured for MPRAGE and was averaged to obtain a mean *D*_MPRAGE_. From four patients scanned with 2D FSE T2 sequence, two lead contact estimates per patient were measured and a total of 8 estimates were averaged to arrive at a mean *D*_2D  FSE  T2_. Four lead contact estimates per patient for each of two patients with 3D FSE T2 sequences, a total of 8 estimates, were averaged and a mean *D*_3D  FSE  T2_ was obtained. Finally, four lead contact estimates per patient for two patients with 2D spin echo T1 results, a total of 8 estimates were averaged and a mean *D*_2D  SE  T1_ was obtained. Six null hypotheses were drawn to test pairwise equivalence of means from the four sequences. A nonparametric statistical test (two-tailed Wilcoxon signed-rank test) was used to draw conclusions about the mean diameter differences at a significance level of 0.05 (Table 1).

## 3. Results

**Figure 2:**
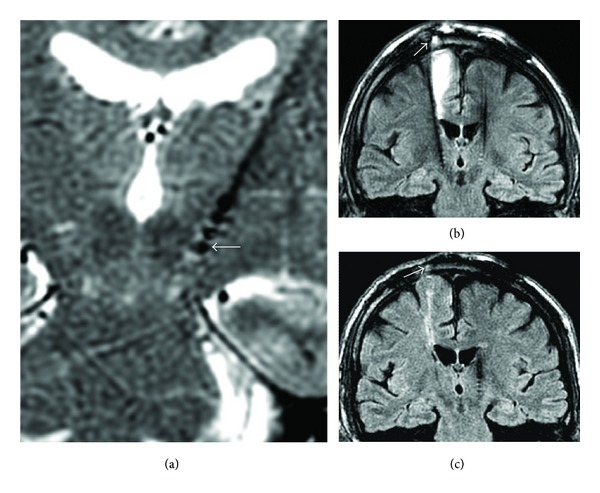
(a) Coronal 3D long echo train T2 image showing 2.2 mm tip diameter (ETL 80, TE 85 ms) with visualization of white matter tissue adequately; (b) and (c) 3D long echo train FLAIR MR signal for an infected DBS lead (arrow showing infection prior to removal (b) and after removal and treatment (c)). Note the artifactual bright dots in T2 images are not prominently present on FLAIR while the size of the implant lead is similar to those in T2 images (Figure 1).

In Figures 1 and 2 typical coronal images from all 4 sequences are shown. In addition the low TE (proton density, Figure 1(c)) and long TE version of 2D FSE (Figure 1(d)) are also compared.

Both the readers (EP, RR) concluded that although the low SAR images are somewhat grainy (due to lower signal-to-noise) cerebral tissue conspicuity away from as well as adjacent to the DBS leads was adequate for radiologic diagnosis with no noticeable quality difference among spin echo, fast spin echo, and MPRAGE sequences. As shown in Figure 2, the FLAIR version of low SAR FSE sequence with long echo train (80–100) and low refocusing flip angles produces similar DBS appearance and is useful for imaging of infections and other complications.

The measured mean lead diameters from the MR images were as follows:

  
*D*_MPRAGE_  = 4.0 ± 0.2 mm,
  
*D*_2D  SE  T1_ = 2.2 ± 0.1 mm,
  
*D*_2D  FSE  T2_  = 2.2 ± 0.1 mm,
 
*D*_3D  FSE  T2_ = 2.1 ± 0.1 mm.

The range of lead contact sizes measured from images for all 8 estimates is listed in Table 1. The mean values represent the limit attainable for each MR sequence with long echo train 3D FSE T2 being the best. A 1.3 mm standard DBS lead contact including perhaps a 0.5 mm encapsulation layer is visualized with the least artifactual signal void of 2.1 mm when 3D FSE T2 MR sequence is used. Two-dimensional FSE T2 at long TE (85 ms) or SE T1 at short TE (14 ms) is also able to restrict the artifactual blooming of DBS contacts and encapsulation layers to a total of 2.2 mm size signal void while short TE (2.3 ms) MPRAGE produces the most artifactual signal loss (4 mm on an average). At longer TE values (T2* weighted gradient echo) the artifacts bloom beyond acceptability for diagnostic use and sequences including susceptibility weighted imaging (SWI) were not tested on DBS recipients.

Note that the range of measured diameters is approximately ±5% of the mean values for all MR sequences. The standard dimensions of the cylindrical Medtronic 3387/3389 DBS electrode contacts are specified as 1.5 ± 0.1 mm height by 1.3 ± 0.1 mm diameter. The electrode encapsulation layer thickness is 0.5 ± 0.4 mm, Haberler et al. [3], Moss et al. [10].

The pairwise statistical mean difference test results are noted in Table 2 and essentially indicate equivalent blooming results for all the 2D sequences while suggesting 3D FSE as the best and 3D MPRAGE as the worst as far as the visualized DBS diameters are concerned. The critical limit attainable to contain the artifactual lead size by any sequence seems to be approximately 2.1 ± 0.1 mm.

## 4. Discussion

To our knowledge, this is the first MR work that utilizes very low RF power on refractory Parkinson's patients to study the lead contact size *in vivo* and relates it to the MRI sequences. The results demonstrate that by lowering echo time or increasing echo train lengths in fast spin echo the tissue visualization surrounding the DBS contacts is better than that obtainable by gradient echo based imaging and it is possible to achieve a fairly small metallic artifact blooming from the DBS leads.

In this work, we assumed the electrode surface is perfectly smooth and ignore any electrode corrosion and surface modification that may occur as a result of implantation process or early stages of stimulation treatment. However, analysis of postmortem or explanted DBS electrodes does not show any visible surface modification of the metallic contacts or tissue changes around the active contact and nonstimulated areas adjacent to the insulated parts as reported by Haberler et al. [3] and Moss et al. [10].

One may expect that the chosen sequence that performs better for lead blooming and tissue contrast would use perfect refocusing RF pulses, long echo train length (ETL), and short echo time (TE). However, in Figure 1, we notice that there is a limit, approximately 2.2 mm diameter of lead size, that is reached even with the minimum TE or using a perfect 180° refocusing condition (as in spin echo T1). The artifact is not reduced when echo train is long, as in routine 2D FSE T2 with 16 echo trains. The use of a very long echo train (80–100) as in 3D FSE T2 MR sequence, even without using perfect 180° refocusing conditions as developed earlier for non-DBS subjects by Sarkar et al. [9], performs the best among the 4 sequences tested. Note that these 3D FSE sequences use optimal, much lower refocusing pulse angles than 180°. One may speculate, based on these results, that the current imaging limit to attain minimum artifactual blooming for MR imaging for a 1.3 mm diameter standard DBS lead contacts surrounded by perhaps a 0.5 mm encapsulation layer is approximately 2.1 mm.

Currently, a number of groups use MRI for assessing DBS lead placement accuracy using high SAR [4] or at ultralow SAR [11, 12]. Three-dimensional MPRAGE is often used to assess the lead placement without directly visualizing the deep brain nuclei that are more conspicuous by FSE sequences. This work shows that for DBS localization there are advantages for using 3D acquisition. With 2D imaging sequences, the slice profiles are less perfect than 3D and often a slice gap is needed to avoid interslice signal contamination. Hence 3D sections are expected to perform better than the 2D counterparts of similar thickness for lead position verification and assessment of adjacent tissue viability in case of suspected complications.

### 4.1. Limitations of the Study

The tissue edge visualization depends on the MR signal and image resolution which directly depend on the magnet field strength, quality of RF coils, and the clinically feasible imaging time. For safety reasons, Parkinson's patients with DBS can currently be imaged only at 1.5 T and with transmit-receive local head coils. Each of these hardware components operates at approximately half the performance level compared to those available for routine patients. Hence refractory PD patients are being imaged at 4-fold lower imaging sensitivity than those imaged at 3 T with multielement head array coils. In addition, due to RF power deposition issues, all of our imaging in this work was done at very low SAR level (0.1 W/kg) that also limits, MR signal-to-noise and extends imaging time. Therefore, with improved hardware and RF safety limits, our results may improve and one may be able to use impedance models and related developments [13] to image substructures around the implant leads in near future more accurately than obtained in this work. Finally, our sample size should be extended to a larger patient population including patients after longer DBS treatments to image the role of and treatment effects on encapsulation layer.

## 5. Conclusions

Conforming to safe RF power limits, this is the first work reporting MRI sequence dependent artifact size of DBS leads in refractory Parkinson's patients and exploring various sequence limits to minimize the artifactual signal void within clinically feasible image resolution and hardware limitations for PD patients. Spin echo and fast spin echo based 2D and 3D FSE sequences provide comparable image blooming of stimulator leads while 3D FSE produces the smallest artifact and the gradient echo based 3D MPRAGE produces the largest.

## Figures and Tables

**Table 1:** The range of lead contact measurements from various MR sequences.

Lead tip-size measurements	*D*_MPRAGE _ (mm)	*D*_2D SE T1 _ (mm)	D_2D FSE T2 _ (mm)	D_3D FSE T2 _ (mm)
1	3.8	2.1	2.3	2.2
2	3.9	2.3	2.1	2.2
3	4.1	2	2.2	2.1
4	4.2	2.2	2	2.1
5	3.9	2.2	2.1	2
6	3.8	2.2	2.1	2
7	3.7	2.1	2.2	2
8	4.3	2.2	2.3	2.1
Mean diameter (mm)	4 ± 0.2	2.2 ± 0.1	2.2 ± 0.1	2.1 ± 0.1

**Table 2:** Wilcoxon signed-rank test results for visualized DBS lead diameters from images using various MR sequences for DBS patients (C.I. *α* = 0.05).

Test number	Null hypotheses mean values (mm)	*W*, *W*_crit_, *P* value	Test results
I.	*H*_0_: (*D*_MPRAGE_ − *D*_2D SE T1_) = 0	0, 3, *P* ≤ 0.05	Reject *H*_0_ (diameters are significantly different)
	4.0 ± 0.2; 2.2 ± 0.1		

II.	*H*_0_: (*D*_MPRAGE_ − *D*_3D FSE T2_) = 0	0, 3, *P* ≤ 0.05	Reject *H*_0_ (diameters are significantly different)
	4.0 ± 0.2; 2.1 ± 0.1		

III.	*H*_0_: (*D*_MPRAGE_ − *D*_2D FSE T2_) = 0	0, 3, *P* ≤ 0.05	Reject *H*_0_ (diameters are significantly different)
	4.0 ± 0.2; 2.2 ± 0.1		

IV.	*H*_0_: (*D*_2D FSE T2_ − *D*_2D SE T1_) = 0	17, 3, *P* ≤ 0.05	Accept *H*_0_ (diameters are not significantly different)
	2.1 ± 0.1; 2.2 ± 0.1		

V.	*H*_0_: (*D*_3D FSE T2_ − *D*_2D SE T1_) = 0	8, 3, *P* ≤ 0.05	Accept *H*_0_ (diameters are not significantly different)
	2.1 ± 0.1; 2.2 ± 0.1		

VI.	*H*_0_: (*D*_3D FSE T2_ − *D*_2D FSE T2_) = 0	8, 3, *P* ≤ 0.05	Accept *H*_0_ (diameters are not significantly different)
	2.1 ± 0.1; 2.2 ± 0.1		

## Abbreviations

PD: Parkinson's disease
SAR: Specific absorption rate
DBS: Deep brain stimulator or stimulation
SE: Spin echo
FSE: Fast spin echo.
